# Multi-step ahead streamflow and uncertainty forecasting using a HyMoLAP rainfall-runoff model-based framework integrated with Bayesian neural networks in the Ouémé river basin, Benin

**DOI:** 10.1371/journal.pone.0333590

**Published:** 2025-10-07

**Authors:** Sianou Ezéckiel Houénafa, Olatunji Johnson, Erick K. Ronoh, Stephen E. Moore

**Affiliations:** 1 Department of Mathematics, Pan African University Institute for Basic Sciences, Technology and Innovation, Nairobi, Kenya; 2 Department of Mathematics, University of Manchester, Manchester, United Kingdom; 3 Department of Agricultural and Biosystems Engineering, Jomo Kenyatta University of Agriculture and Technology, Nairobi, Kenya; 4 Department of Mathematics, University of Cape Coast, Cape Coast, Ghana; 5 Center for Artificial Intelligence Research, Cape Coast, Ghana; Swedish Meteorological and Hydrological Institute, SWEDEN

## Abstract

Multi-step forecasting is crucial for capturing future streamflow variations and managing water resources but remains challenging due to limited accuracy of upstream flow forecasts and meteorological predictions over lead times. While data-driven methods are commonly used, this study extends the Hydrological Model based on the Least Action Principle (HyMoLAP) from daily rainfall-runoff simulation to multi-day-ahead streamflow predictions. Additionally, it integrates Bayesian Long Short-Term Memory (Bayesian LSTM), primarily to enable uncertainty quantification (UQ). Applied to the Bonou and Savè sub-catchments of the Ouémé River Basin, Benin, the HyMoLAP-based framework yields NSE values ranging from 0.997 to 0.921 at Bonou and from 0.970 to 0.799 at Savè, showing slightly higher performance than the LSTM model overall, except at Savè from the 3-day lead time onward where it becomes slightly lower, with a more pronounced difference at the 7-day horizon. Our UQ approach provides reliable prediction intervals, with a coverage probability around 90%, as nearly 90% of the observed data fall within the 90% credible intervals in both sub-catchments.

## 1 Introduction

Many hydrological applications related to water resource planning and management primarily rely on a succession of runoff forecasts with extensive lead times [1]. Multi-step-ahead forecasting stands to be a crucial approach for anticipating future streamflow variations, enabling effective decision-making in flood management, reservoir operations, and drought mitigation. Accurate medium- to long-term runoff forecasting is of great significance for flood control, drought mitigation, comprehensive water resource management, and ecological restoration [2]. One-step-ahead forecasting is also of practical and scientific interest [3]. However, due to the strong correlation between streamflow and other hydrological variables, such as precipitation, multi-step-ahead forecasting becomes more challenging, as it requires capturing complex dependencies over extended time horizons. Since no information is available about future conditions at the time of prediction, the models must rely on past data and its previously predicted values, making it difficult to capture significant variations and maintain accuracy over extended lead times.

Streamflow forecasting methods are typically classified into three main categories: physically based models, conceptual models, and empirical models [4]. In the context of streamflow simulation, physically based models facilitate the simulation process by solving differential equations that govern the movement of water. Examples of such models include the Systeme Hydrologique European (SHE) [5], the Institute of Hydrology Distributed Model (IHDM) [6], and the Hydrological Model based on the Least Action Principle (HyMoLAP) [7–9]. The HyMoLAP model has been applied for various tasks, including daily rainfall-runoff simulation [10,11] and uncertainty analysis through its reformulation as stochastic differential equations [12,13], demonstrating its strong predictive capabilities. Empirical models, also known as data-driven models, have been widely employed in hydrological modeling [14–20]. These models operate as computational frameworks that establish relationships between input and output data without accounting for the underlying physical processes [21]. Examples of such models include Autoregressive Integrated Moving Average (ARIMA) [22], Random Forest [23], and Artificial Neural Networks (ANNs) [24]. A notable advantage of data-driven models is their flexible structure, which facilitates integration with process-oriented models (conceptual and physically based), leading to hybrid approaches, as illustrated in recent studies [25,26]. Simply put, these hybrid models combine the strengths of both process-oriented and data-driven approaches [27]. They have been widely employed in hydrology to achieve improved accuracy and more reliable predictions, as demonstrated in [28–31].

Deep learning models, a subset of machine learning, equipped with multiple hidden layers have been proposed and successfully applied to streamflow forecasting, as shown in [32–35]. They have been prominent in environmental and climate change problems [36]. These models rely on ANNs, which are designed to automatically extract hierarchical features from data, making them particularly effective for capturing complex temporal dependencies. A Recurrent neural network (RNN) is a type of sequence model that maintains a vector of hidden activation that propagates over time [37]. However, traditional RNNs face difficulties in capturing long-range dependencies, often suffering from the vanishing gradient problem [38]. To address this limitation, Long Short-Term Memory (LSTM) networks were introduced, incorporating specialized gating mechanisms to regulate the flow of information. These gates help preserve important features over long sequences, effectively mitigating the vanishing gradient problem. To improve computational efficiency and reduce memory usage, a streamlined variant of LSTM called Gated Recurrent Unit (GRU) was introduced by [39]. It simplifies the internal structure of the model while retaining the ability to capture both short- and long-term dependencies in sequential data. The LSTM model has been widely used for streamflow simulation, demonstrating its superiority over many other models for this task, as shown in [40,41]. This model has been applied in various contexts, such as rainfall-runoff simulation, where only meteorological data are used to estimate runoff [42], and multi-step streamflow forecasting [43]. Additionally, it has been hybridized with hydrological models to enhance simulation accuracy, as demonstrated in [19,44].

In hydrology, uncertainties are ever-present, driving scientists and engineers to continuously improve estimation and mitigation techniques [45]. To enhance risk-based decision-making in water resource management, existing streamflow forecasting methods should be improved to estimate uncertainties in their predictions [46]. The hydrological sciences community has put substantial effort into developing methods for providing uncertainty estimations around traditional models [47]. The understanding and quantification of uncertainties in the process should evolve together with the advancements in modeling techniques, such as machine learning (ML), Artificial Intelligence (AI), for the better use of the available datasets [48]. In recent years, Bayesian Neural Networks (BNNs) [49] have gained attention for their ability to quantify uncertainty in deep learning models. By incorporating probabilistic representations of weights, BNNs provide more robust and interpretable predictions, making them suitable for uncertainty forecasting. In particular, it was demonstrated in [50] that Bayesian LSTM can be effectively used for Uncertainty Quantification (UQ) in streamflow modeling. While the study emphasizes its potential for data-scarce catchments, the validation was carried out in both snowmelt-dominated (East River, Colorado) and rainfall-dominated (Alabama–Coosa–Tallapoosa, southeastern U.S.) basins.

Data-driven techniques, as demonstrated by [3,43,51,52], are the common models used for multi-step-ahead predictions. These models offer flexibility and computational efficiency in hydrological predictions. However, as noted in [53], one of the main challenges with multi-timescale DL-based hydrological forecasting is the potential inconsistency (discrepancy) between forecasts across different timescales. This issue is often manifested in the accumulation of errors, which becomes particularly evident in long-term forecasts. This is an expected outcome given that the observed data is limited to the start date of the forecasts. This led to the examination of the issue of error evolution in multi-step-ahead hydrological forecasting by [54]. For effective water resource management, several multi-step forecasting methods should be recommended in order to conduct comparative studies and identify those that offer the best performance.

This study proposes a multi-step streamflow forecasting framework for the Ouémé River Basin by extending the physically based HyMoLAP model, which is particularly well suited to this catchment thanks to its parsimonious structure and minimal data requirements. Unlike many hydrological models, HyMoLAP is formulated as a simple ordinary differential equation (ODE), which not only facilitates calibration under data-scarce conditions and generates corresponding daily streamflow from only meteorological data, but can also explicitly incorporates previous days’ streamflows through the chosen numerical discretization scheme to predict the day ahead—providing a distinctive advantage to the proposed framework. To enhance the framework capability and extend it to uncertainty quantification, we develop a hybrid model that combines HyMoLAP with a Bayesian LSTM. Specifically, by applying both implicit and explicit Euler-Maruyama schemes, the HyMoLAP model uses the explicit scheme for 1-day ahead forecasting, and we then derive a recurrent relation for multi-day predictions. The resulting streamflow simulations serve as input to the Bayesian LSTM, which mainly quantifies uncertainty through Bayesian inference.

The rest of the paper is organized as follows: Sect 2 reviews related works on multi-step streamflow modeling. Sect 3 presents the materials and research methods. Sect 4 presents and discusses the results. Finally, Sect 5 concludes the paper.

## 2 Related work

Various time series analysis models and machine learning methods, such as Autoregressive (AR), Moving Average (MA), Autoregressive Moving Average (ARMA), and Autoregressive Fractionally Integrated Moving Average (ARFIMA), alongside Neural Networks (NN), Random Forests (RF), and Support Vector Machines (SVM), were evaluated in [3] for multi-step-ahead forecasting of hydrological processes through large-scale computational experiments. The study, based on simulations assuming linearity and stationarity, demonstrated that both approaches are equally effective for univariate short-time series forecasting over extended time horizons. In [55], a spatial deep learning framework, known as the directed graph deep neural network, was introduced for multi-step streamflow forecasting. In addition to enhancing predictive accuracy, a hidden Markov regression model was integrated to assess the uncertainty associated with its forecasts. Moreover, the study conducted in [1] presents two novel architectures, DirCNN and DRCNN, designed for multi-step-ahead monthly streamflow prediction. These approaches adopt direct (Dir) and direct-recursive (DR) forecasting strategies while leveraging convolutional neural networks (CNNs) for automatic feature extraction. Multi-step-ahead monthly streamflow prediction was also approached in [56] using a neurofuzzy network model and in [51] using a long short-term memory (LSTM) network. The research conducted by [57] evaluated the performance of four distinct models—artificial neural networks (ANNs), support vector regression (SVR), wavelet-ANN, and wavelet-SVR—across Mediterranean, Oceanic, and Hemiboreal watersheds for forecasting 1-, 2- and 3-day ahead streamflow. While SVR-based models demonstrated the highest overall performance under the study’s assumptions, no single model consistently outperformed the others in more than one watershed. This finding suggests that the effectiveness of a given model may depend on the specific characteristics of the data. Different combinations of climatic and hydrological variables were used in [58] as inputs for three AI-based models—LSTM, gated recurrent unit (GRU), and least-squares support vector machine (LSSVM)—to predict short-term streamflow. This study highlights the potential of AI-based approaches for multi-step streamflow forecasting, as well as the study conducted by [51]. A new, simpler model combining Random Forest and Multilayer Perceptron through a stacking approach, with the Elastic Net algorithm as a meta-learner, was introduced in [59] for multi-step-ahead streamflow forecasting. Additionally, a more complex model based on bi-directional Long Short-Term Memory (LSTM) networks was also employed for comparison. A Three-Step LSTM model enhancement was introduced in [60] for multi-step streamflow forecasting. In Step 1, LSTM outperformed a conventional ANN baseline (*R* = 0.9055, MSE=17.8532, MAE=1.4365, NSE=0.8190, RMSE=5.3695). Step 2, incorporating the rate-of-change model, improved performance (*R* = 0.9545, MSE=8.9746, MAE=0.5434, NSE=0.9090, RMSE=2.9958). Finally, Step 3 (bat-LSTM hybrid) achieved the best results (*R* = 0.9757, MSE=4.7187, MAE=0.4672, NSE=0.9514, RMSE=2.1723). In [61], LSTM and Multi-Layer Perceptron (MLP) models, based on rates of change, were employed for multi-step ahead streamflow forecasting. Reservoir computing (RC) methods, optimized using the Coyote optimization algorithm, have been explored [62] for one, seven, fourteen, and twenty-one steps ahead streamflow prediction, demonstrating improved accuracy when combined with time series decomposition techniques such as variational mode decomposition (VMD) and empirical wavelet transform. The authors in [53] proposed an hierarchical deep learning (HDL) model, which integrates temporal hierarchical reconciliation (THR) with deep learning for seven-day-ahead streamflow forecasting, providing a novel and generalizable framework for consistent multi-timescale water resources prediction. Furthermore, Bayesian Deep Learning replaces the weight parameters of deterministic networks with probability distribution over these parameters, and instead of optimizing the network weights to find a single set of values that best fit the training data, it considers all possible weights that are likely to have generated the data [50]. It has been applied to multi-step uncertainty quantification, as demonstrated in studies by [63–66].

Recent advances in artificial intelligence have led to the development of more sophisticated models, such as Transformers, Kolmogorov–Arnold Networks, and their variants. A growing number of these approaches have been applied in hydrology for multi-step forecasting, although their use remains relatively limited. In [67], a Transformer-based deep learning approach was employed for multi-step ahead daily streamflow forecasting, with performance evaluated across different scenarios. Similarly, the authors of [68] proposed SMGformer, a deep learning model that integrates Seasonal and Trend decomposition using Loess (STL) with multi-head self-attention for multi-step runoff forecasting. In [52], an MVMD-ensembled Transformer model (MVMD-Transformer) was introduced, incorporating the MVMD for concurrent time–frequency analysis of streamflow and related potential influencing variables. A hybrid model combining convolutional neural networks (CNN), Transformers, and LSTM networks (CTL) was developed, as reported in [69], and it outperformed all standalone models in terms of predictive accuracy. Moreover, the authors in [70] introduced a coupled model, CNN-LSTM-Self-Attention Anticipated Learning Machine (CLS-ALM), which integrates nonlinear dynamical systems with deep learning techniques for multi-step runoff forecasting. On the other hand, the study by [71] evaluated the performance of two advanced deep learning models—Kolmogorov–Arnold Networks (KAN) and Transformers—using long-term hydrological data from four major Central European rivers. The results showed that the KAN significantly outperformed the Transformer in short-term forecasts (up to 3 days). However, as the forecasting horizon extended to 7 days, the performance of both models converged. Moreover, the authors in [72] evaluated three machine learning models—Temporal Kolmogorov–Arnold Networks (TKAN), LSTM, and Temporal Convolutional Networks (TCN)—with a focus on their ability to improve prediction accuracy and efficiency in streamflow forecasting. For multi-step forecasting, the results showed that TKAN exhibited strong performance up to a three-day forecast horizon, with only a slight decline in accuracy as the forecasting period extended. Collectively, these studies underscore the potential of advanced ML methods to improve the accuracy and reliability of multi-step streamflow forecasting.

Similarly to the above methods, numerous other data-driven approaches have been developed for multi-step streamflow forecasting, as demonstrated by the studies in [73–86]. It can be observed that multi-step ahead streamflow forecasting methods are data-driven approaches. Since multi-step predictions are crucial for analyzing extended periods, developing different methods for comparison remains of great interest, as no single model is universally optimal for all hydrological systems. In contrast, hydrological models offer a structured framework that can drive improvements by integrating physical constraints and enhancing interpretability. In this context, our work demonstrates that, in the Ouémé catchment, multi-step ahead forecasting can be effectively achieved using hydrological models, specifically the Hydrological Model based on the Least Action Principle (HyMoLAP). We build on HyMoLAP to forecast multi-day ahead streamflow in two sub-catchments within the Ouémé basin, and compare its performance with the LSTM model’s multi-step predictions. To further enhance the framework, we propose a hybrid model where the simulated multi-step streamflow from HyMoLAP serves as input to a Bayesian LSTM, enabling uncertainty estimation through hybridization.

## 3 Materials and methods

### 3.1 Hydrological Model based on the Least Action Principle (HyMoLAP)

The Hydrological Model based on the Least Action Principle (HyMoLAP) is a physically-based rainfall-runoff model formulated as a dynamical system. Initiated in [7] and later developed in [8,9], the formulation of the model is grounded in the Least Action Principle (LAP), a fundamental concept in physics further refined by Noether’s theorem [87]. This principle was analytically formulated, resulting in the representation of rainfall-runoff systems as an Ordinary Differential Equation (ODE):

$$\frac{dQ(t)}{dt}=-\frac{\mu }{\lambda }Q(t{)}^{2\mu -1}+\frac{1}{\lambda }\psi (q(t),t),\;\text{with}\;Q(t_{0})=Q(t=0),$$ (1)

where *Q*(*t*) represents the discharge at time *t*, and *t*_0_ is the initial day. *μ* and *λ* are the two parameters of the model. They represent the nonlinearity parameter and the macroscopic parameter, respectively, describing emergent properties related to the geomorphology and pedology of the river basin [9]. ψ(q(t),t) is a composite forcing function that describes the input or forcing of the model.

This study utilizes a simple formulation of ψ(q(t),t). Its common definition is ψ(q(t),t)=x(t)q(t), where q(t)=max(0,P(t)−PET(t)) represents the effective precipitation, with *P*(*t*) denoting precipitation and *PET*(*t*) denoting potential evapotranspiration. Moreover, *x*(*t*) is a proportionality variable that describes the state of the basin [9], particularly its moisture content. With Δt=1 day, aligning with the daily timestep of the data, a discrete form of the HyMoLAP model using the explicit Euler method is:

$$Q_{t+1}=Q_{t}-\frac{\mu }{\lambda }Q_{t}^{2\mu -1}+\frac{1}{\lambda }x_{t}q_{t},\;\text{with}\;Q_{0}=Q(t=0),$$ (2)

where $q_{t}=\max(0,P_{t}-PET_{t})$ and *x*_*t*_ satisfies the following discrete dynamical system for t = 1,2,3,...:

$$x_{t}=\left\{ \begin{array}{cc} x_{t-1}-\frac{\mu }{\lambda }x_{t-1}, & \text{if }q_{t}=0\\ x_{t-1}+\frac{\mu }{\lambda }q_{t}, & \text{if }q_{t}>0 \end{array}\right..$$ (3)

The implementation of the model requires an initial value of *x*_*t*_ which describes the initial state of the basin. However, in this paper, we used an estimated $x_{0}=P_{0}-PET_{0}$, as employed in [88].

### 3.2 Long-Short Term Memory (LSTM)

Introduced by [89], LSTM is one of the most widely used deep learning models with strong long data processing capability [90]. Given a time series $X_{t}=(x_{1},x_{2},\ldots {)}_{t}$, the LSTM hidden state *h*_*t*_ at step *t* is updated through four components—the forget gate *f*(*t*), input gate *i*_(*t*)_, output gate *o*_(*t*)_, and cell state *c*_(*t*)_. The forward pass of an LSTM cell can be compactly written as:

$$f_{\left(t\right)}=\sigma (U_{f}\cdot h_{\left(t-1\right)}+W_{f}\cdot X_{\left(t\right)}+b_{f})$$ (4)

$$i_{\left(t\right)}=\sigma (U_{i}\cdot h_{\left(t-1\right)}+W_{i}\cdot X_{\left(t\right)}+b_{i})$$ (5)

$$o_{\left(t\right)}=\sigma (U_{o}\cdot h_{\left(t-1\right)}+W_{o}\cdot X_{\left(t\right)}+b_{o})$$ (6)

$${\tilde{c}}_{\left(t\right)}=\tanh(U_{c}\cdot h_{\left(t-1\right)}+W_{c}\cdot X_{\left(t\right)}+b_{c})$$ (7)

$$c_{\left(t\right)}=f_{\left(t\right)}⊙c_{\left(t-1\right)}+i_{\left(t\right)}⊙{\tilde{c}}_{\left(t\right)}$$ (8)

$$h_{\left(t\right)}=o_{\left(t\right)}⊙\tanh(c_{\left(t\right)}),$$ (9)

where *U*, *W* and *b* are respectively the weight matrices and bias vector parameters which need to be learned during training, *σ* is the sigmoid activation function, ⊙ represents the element-wise Hadamard product and tanh is the hyperbolic tangent activation function. ${\tilde{c}}_{\left(t\right)}$ represents the candidate cell state at time step *t*, and the outputs of the input and forget gates are used to update the cell state *c*_(*t*)_ and the current hidden state *h*_(*t*)_. These six equations describe how an LSTM cell processes input data while preserving long-term dependencies through the cell state.

LSTM has been widely applied in hydrology to enhance the modeling and prediction of rainfall–runoff processes and streamflow variations [42,91,92]. For long lead times, LSTM has shown strong ability to capture streamflow variation over extended periods, outperforming conventional ANNs [60]. It has also been integrated into hybrid frameworks with other ML models, such as CNNs and transformers, for long-term prediction, yielding significant improvements [69].

### 3.3 Bayesian Neural Network

A crucial element often missing is a clear understanding of the uncertainties associated with the dataset [93]. Bayesian Neural Networks (BNNs) integrate uncertainty into deep learning models by adopting a Bayesian framework. Unlike traditional neural networks that estimate a single set of fixed parameters, BNNs assign a probability distribution to these parameters and seek to infer their posterior distribution [94].

It was demonstrated in [95] that dropout can be interpreted as a variational approximation of the posterior distribution in a Bayesian neural network. Specifically, using a mixture of two Gaussian distributions as the variational distribution, dropout training minimizes the Kullback-Leibler (KL) divergence [96] between an approximate posterior and the true posterior. With Bernoulli noise being the most commonly applied type, dropout functions by introducing multiplicative noise to the target layer [49]. Dropout is typically disabled during evaluation, but keeping it active allows for obtaining a distribution of predictions rather than a single deterministic value. This approach, known as Monte Carlo Dropout (MC dropout), enables the estimation of prediction uncertainty by performing multiple forward passes on the same input. By applying this formulation, MC dropout training serves as a variational approximation of Bayesian inference, encouraging a distribution over weights rather than relying on point estimates. While this approach can mitigate overfitting, dropout in a Bayesian setting should not be considered a regularization technique, as it forms part of the variational posterior rather than acting as a prior constraint. Consequently, MC Dropout enables efficient Bayesian inference, allowing for uncertainty estimation in deep learning models without requiring explicit posterior sampling techniques.

This study follows the MC dropout framework as shown in [94], which enables uncertainty estimation without modifying the model architecture and incurs minimal computational overhead. By applying dropout at inference time, each forward pass through the network samples a different realization of the weights, effectively approximating a Bayesian LSTM. Model uncertainty was quantified by computing the credible interval (CrI) of the predictions, given by:

$$\text{CrI}=\bar{\hat{y}}\pm z_{\alpha /2}\cdot \sigma_{\hat{y}},$$ (10)

where $\bar{\hat{y}}$ is the mean prediction, $\sigma_{\hat{y}}$ is the standard deviation of predictions across multiple runs, and $z_{\alpha /2}$ is the critical value for a confidence level 1−α.

It is important to note that the MC dropout framework is widely valued by practitioners for its versatility, ease of implementation, and seamless integration with existing neural networks, but its application to real-world problems remains largely unexplored [94].

### 3.4 HyMoLAP-based framework and hybrid HyMoLAP-Bayesian LSTM for multi-Step forecasting

An ordinary differential equation can be solved using various numerical techniques, and the Euler method [97] is one of them. This method can be either explicit or implicit. Applying the Euler technique to the HyMoLAP model, we recall the explicit form that we show in Eq (11), while an implicit form is given in Eq (12):

$$Q_{t+1}=Q_{t}-\frac{\mu }{\lambda }Q_{t}^{2\mu -1}+\frac{1}{\lambda }\psi_{q_{t},t},$$ (11)

$$Q_{t+1}=Q_{t}-\frac{\mu }{\lambda }Q_{t+1}^{2\mu -1}+\frac{1}{\lambda }\psi_{q_{t+1},t+1}.$$ (12)

These two discretized Eqs (11)–(12) can be interpreted differently in hydrological modeling. Indeed, given an initial discharge *Q*_0_, observed precipitation *P* and potential evapotranspiration *PET* data, we have:

- Eq (11) predicts the one-day-ahead runoff using precipitation and potential evapotranspiration data.
- Eq (12) generates the corresponding daily runoff using the same meteorological data.

The second case (Eq (12)) aligns with the approach used in models such as Génie Rural à 4 paramètres Journalier (GR4J) [98], Génie Rural à 6 paramètres Journalier (GR6J) [99], and Hydrologiska Byråns Vattenbalansavdelning (HBV) [100], where the corresponding daily runoff is generated based solely on meteorological data.

Now, in our framework, given any initial multi-step prediction day *t* with the observed *Q*_*t*_, *P*_*t*_ and *PET*_*t*_, the explicit Eq (11) allows us to predict the streamflow for *t*  +  1. So, for a *t*  +  2 prediction, the same Eq (11) becomes:

$$Q_{t+2}=Q_{t+1}-\frac{\mu }{\lambda }Q_{t+1}^{2\mu -1}+\frac{1}{\lambda }\psi_{q_{t+1},t+1}.$$ (13)

Since we need $\psi_{q_{t+1},t+1}$ in the above Eq (13) to predict *Q*_*t* + 2_, we used the implicit Eq (12) to approximate it, as shown in the following expression:

$$\psi_{q_{t+1},t+1}=\lambda \left(Q_{t+1}-Q_{t}+\frac{\mu }{\lambda }Q_{t+1}^{2\mu -1}\right).$$ (14)

Substituting Eq (14) in Eq (13) and following the same reasoning, we obtain the following relations for 1- to *T*-day ahead predictions:

$${\hat{Q}}_{t+1}=Q_{t}-\frac{\mu }{\lambda }Q_{t}^{2\mu -1}+\frac{1}{\lambda }\psi_{q_{t},t},$$ (15)

$${\hat{Q}}_{t+2}=2*{\hat{Q}}_{t+1}-Q_{t},$$ (16)

$${\hat{Q}}_{t+3}=2*{\hat{Q}}_{t+2}-{\hat{Q}}_{t+1},$$ (17)

⋮ (18)

$${\hat{Q}}_{T}=2*{\hat{Q}}_{T-1}-{\hat{Q}}_{T-2},$$ (19)

with $\hat{Q}$ representing the predicted streamflow and *T* denoting the final time of the multi-step prediction. Note that here, once the HyMoLAP parameters *μ* and *λ* in Eq (15) are calibrated, they remain fixed throughout the simulation and are not re-calibrated for each forecast horizon.

The main difference between this framework and the role of HyMoLAP in the literature is that here, we predict discharge for multiple consecutive days ahead using the observed discharge, precipitation, and evapotranspiration on the initial multi-step prediction day, whereas HyMoLAP is typically designed for daily rainfall-runoff modeling, simulating daily discharge solely from observed daily precipitation and evapotranspiration. It is worth noting that the above recurrent Eqs (16)–(19), imply that the difference in streamflow between two consecutive time steps remains constant over a certain period *T*, without necessarily meaning that the contribution of climatic variables remains unchanged. Beyond 1-day lead time, this assumption may appear overly simplistic in basins subject to strong precipitation pulses, where sudden rainfall events can induce abrupt variations in streamflow that a constant-difference representation fails to capture. In such cases, the formulation acts only as a local approximation that smooths rapid fluctuations. However, this approach can introduce errors that accumulate over time, since each prediction is reused in subsequent steps. For long lead times, the error accumulation can significantly lead to overall accuracy issues, a challenge also observed in data-driven models for multi-step forecasting [64]. To improve the framework, we then integrate the above with a Bayesian LSTM model. Indeed, a key advantage of Bayesian neural networks is that their uncertainty estimates align more closely with observed errors, making them less prone to overconfidence or underconfidence [49]. This approach can enhance point predictions and, more importantly, enables interval predictions, which are crucial for reliable multi-step probabilistic streamflow forecasting.

In [50], it was highlighted that combining various types of hydrological data with the LSTM network in different ways can facilitate the creation of hybrid models, such as using the LSTM to learn the residuals from physics-based model simulations, or incorporating hydrological principles into the LSTM loss function. The latter example describes the classic concept of Physics-Informed Neural Networks (PINNs) [101]. These methods benefit from both the physical model and the neural network. However, another common approach to hybridizing hydrological models with data-driven methods involves using simulated processes or outputs from hydrological models as inputs for data-driven techniques, as shown in [31,44,50,88,102].

**Fig 1:**
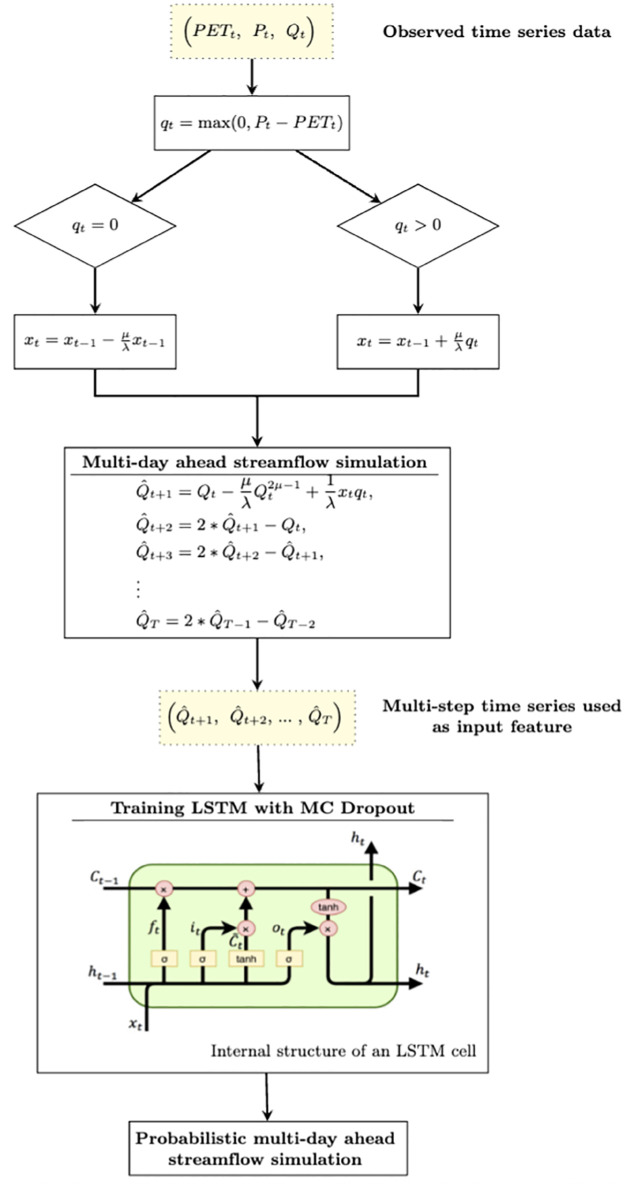
Architecture of the HyMoLAP–Bayesian LSTM hybrid model for multi-step forecasting.

This form of hybridization can also be extended to uncertainty quantification, where simulations from hydrological models serve as inputs to a Bayesian LSTM model. Consequently, for uncertainty quantification in this study, we follow this approach by incorporating the multi-step discharge simulated by the framework based on HyMoLAP model described above as the sole input for the Bayesian LSTM model. We acknowledge that combining simulated discharge with meteorological data might improve predictive accuracy at longer lead times. However, our choice is guided by the assumption that meteorological forecasts are not available and the restriction against using observed data as predictors. In this context, the recurrent formulation is particularly valuable, as HyMoLAP can be directly applied to simulate runoff using precipitation and evapotranspiration as the available inputs. This approach is consistent with the LSTM simulation setup in this study, which likewise relies solely on previously predicted streamflow for long-term forecasting. For the hybrid model, during inference, multiple stochastic forward passes are performed to generate prediction intervals, effectively capturing uncertainty in multi-step ahead streamflow forecasts. This approach allows for the capture of both the inherent uncertainty in the multi-step prediction and the temporal dependencies present in the discharge data, providing a novel and robust framework for probabilistic multi-step discharge prediction. Fig 1 presents the structure of the HyMoLAP–Bayesian LSTM hybrid model. Notably, this architecture incorporates the multi-step point simulation approach derived from the HyMoLAP model, starting from the beginning up to the *Multi-day ahead streamflow simulation* step indicated in the figure. We provide the Python simulation code, implemented with the PyTorch deep learning library, as part of the GitHub repository: https://github.com/Ezesia-lab/HyMoLAP_Bayesian_LSTM.

### 3.5 Evaluation criteria

For the objective function used in evaluating hydrologic model performance, it is beneficial to incorporate multiple metrics that capture various aspects of the model’s effectiveness [103]. In this study, for accuracy evaluation, we employed the Nash–Sutcliffe Efficiency (NSE) [104], the Root Mean Square Error (RMSE) [105], and the Kling-Gupta Efficiency (KGE) [106]. Furthermore, to assess the reliability and sharpness of prediction intervals, we used the Prediction Interval Coverage Probability (PICP) and the Mean Prediction Interval Width (MPIW) [64]. The PICP is the proportion of instances where the prediction interval contains the true value [107]. The MPIW measures the average width of these intervals. In accordance with the interval prediction guidelines outlined in [108], a desirable uncertainty quantification result should exhibit a high PICP and a low MPIW value. These metrics are mathematically expressed as follows:

$$\text{NSE}=1-\frac{\sum_{i=1}^{N}(Q_{i}-{\hat{Q}}_{i}{)}^{2}}{\sum_{i=1}^{N}(Q_{i}-\bar{Q}{)}^{2}},$$ (20)

$$\text{RMSE}=\sqrt{\frac{1}{N}\sum_{i=1}^{N}(Q_{i}-{\hat{Q}}_{i}{)}^{2}}\;(m^{3}/s),$$ (21)

$$\text{KGE}=1-\sqrt{(r-1{)}^{2}+{\left(\frac{\sigma_{Q}}{\sigma_{\hat{Q}}}-1\right)}^{2}+{\left(\frac{\mu_{Q}}{\mu_{\hat{Q}}}-1\right)}^{2}},$$ (22)

$$\text{PICP}=\frac{1}{N}\sum_{i=1}^{N}𝕀\left[{\hat{Q}}_{i}^{\text{lower}}\leq Q_{i}\leq {\hat{Q}}_{i}^{\text{upper}}\right],$$ (23)

$$\text{MPIW}=\frac{1}{N}\sum_{i=1}^{N}\left({\hat{Q}}_{i}^{\text{upper}}-{\hat{Q}}_{i}^{\text{lower}}\right),$$ (24)

where *N* represents the total number of data points. *Q*_*i*_ is the observed discharge at time step *i*, and ${\hat{Q}}_{i}$ is the predicted discharge at the same time step. $\bar{Q}$ is the mean of the observed discharges, while $\mu_{Q}$ and $\mu_{\hat{Q}}$ are the means of the observed and predicted discharges, respectively. The standard deviations of the observed and predicted discharges are represented by $\sigma_{Q}$ and $\sigma_{\hat{Q}}$, respectively. Furthermore, *r* refers to the Pearson correlation coefficient [109], which quantifies the degree of correlation between the observed and predicted discharges. Finally, ${\hat{Q}}_{i}^{\text{lower}}$ and ${\hat{Q}}_{i}^{\text{upper}}$ denote the lower and upper limits of the prediction interval for the observation of *i* the -th, respectively, and 𝕀[·] is the indicator function.

### 3.6 Study area and data

**Fig 2:**
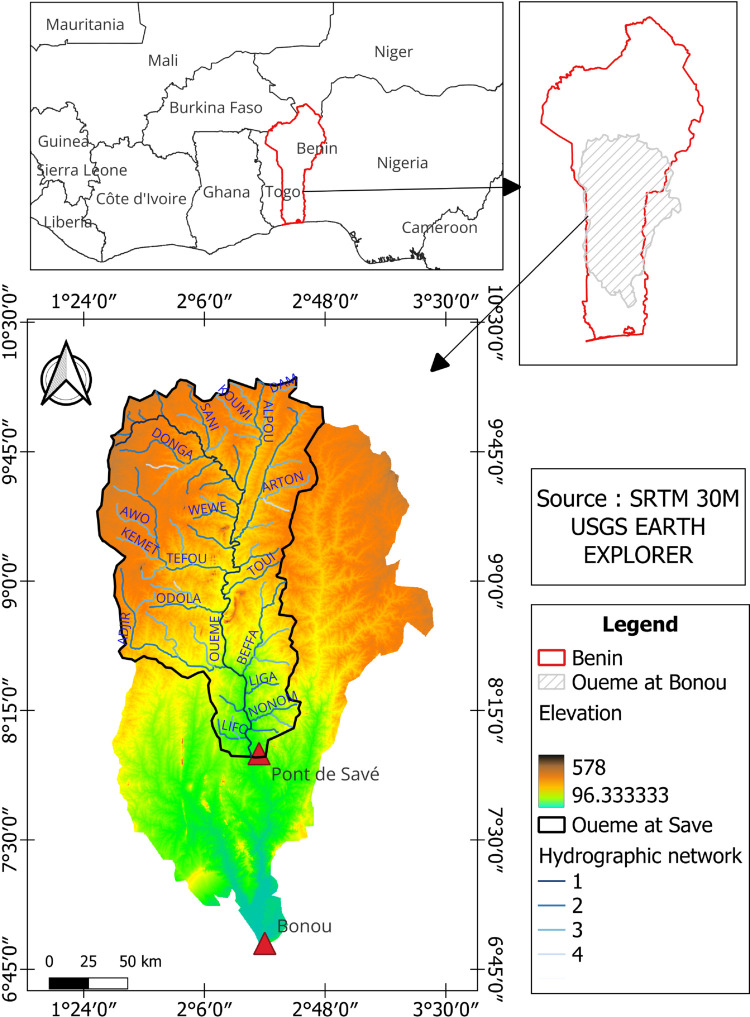
The location and main characteristics of the study area. The red triangles indicate the hydrometric stations for the Bonou and Savè sub-catchments. Map generated by the authors using SRTM 30m digital elevation data (USGS Earth Explorer, public domain).

**Fig 3:**
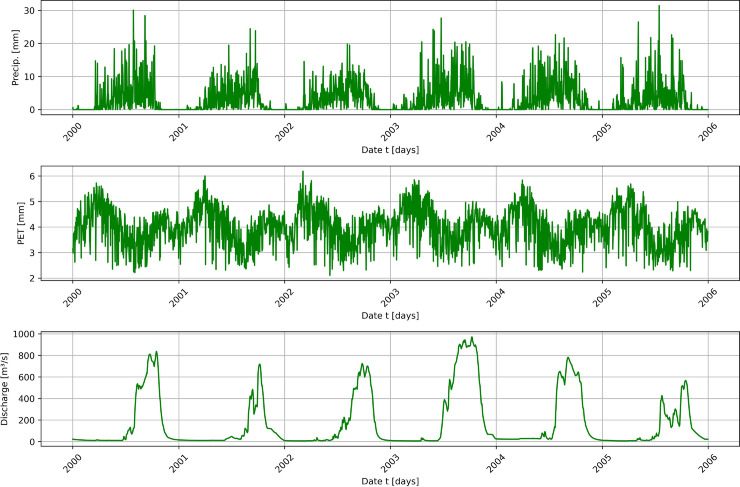
Daily time series plot of precipitation, potential evapotranspiration (PET), and discharge in the Bonou sub-catchment.

**Fig 4:**
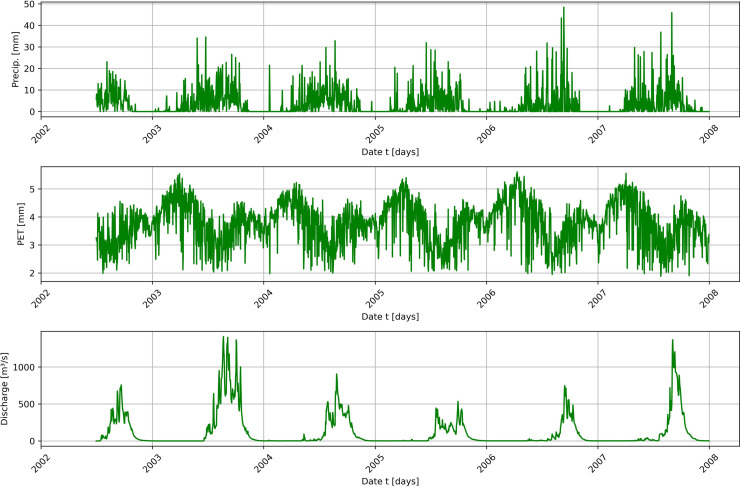
Daily time series plot of precipitation, potential evapotranspiration (PET), and discharge in the Savè sub-catchment.

Situated in the Republic of Benin, the Ouémé River Basin encompasses nearly half of the country’s total area [110]. It spans two distinct climatic zones: the Guinea savanna zone and the Soudanese savanna zone [12]. This study specifically focuses on the Bonou and Savè sub-catchments within the Ouémé river basin (Fig 2). The Savè sub-catchment ($\approx 23,600\;{km}^{2}$) is about half the size of the Bonou sub-catchment ($\approx 48,900\;{km}^{2}$). Daily data on discharge, precipitation, and potential evapotranspiration were used in this study, as depicted in Figs 3 and 4. Precipitation, which is is an essential factor affecting runoff [111], and potential evapotranspiration data were supplied by Météo-Benin, while discharge data were obtained from the National Directorate of Water (DG-Eau), Benin. It is important to note that discharge values exhibit rapid fluctuations from one season to another in both sub-catchments. These variations demonstrate clear seasonality, with peaks corresponding to high precipitation events, as illustrated in the figures.

In the LSTM and hybrid HyMoLAP–Bayesian LSTM hybrid models, the MinMax normalization technique, commonly employed in data-driven modeling, was applied. This choice, instead of z-score standardization, was motivated by the high seasonal variability of discharge data. MinMax scaling preserves distribution shape and temporal variability, whereas z-score may distort extremes and seasonal peaks. In fact, normalization in deep learning plays a vital role in ensuring that all input features are brought to a uniform scale. Moreover, it helps maintain the robustness of optimization methods like gradient descent, allowing them to converge more effectively to the optimal solution. In this research, daily streamflow data and associated predictors were rescaled to lie within the interval [0, 1] using the MinMax normalization technique, as specified in Eq (25), prior to conducting forecasts:

$$X_{\text{scaled}}=\frac{X-X_{\text{min}}}{X_{\text{max}}-X_{\text{min}}},$$ (25)

where *X* is the original value, $X_{\text{min}}$ and $X_{\text{max}}$ are the minimum and maximum values of the dataset, respectively, and $X_{\text{scaled}}$ is the normalized value ranging between 0 and 1.

For the Bonou sub-catchment, two-thirds of the data (2000–2003, approximately 66.66%) were used for calibration, while one-third (2004–2005, approximately 33.33%) was allocated for validation. In the case of Savè, calibration covered seven-elevenths of the data (mid-2002 to 2005, approximately 63.63%), while the remaining four-elevenths (2006–2007, approximately 36.37%) were used for validation. This setup ensured a two-year validation period for both sub-catchments.

### 3.7 Experiment design

Model calibration is the systematic tuning and assessment of the most influential and sensitive parameters until the model outputs closely align with the observed behavior of the measured system within a basin [112]. The calibration process of the HyMoLAP model in this study involves estimating the structural function using a heuristic approach based on NSE criteria and the explicit Euler discretization method. The heuristic approach consists of the following steps: (1) Defining initial intervals (large intervals) for *μ* and *λ*; (2) Randomly selecting values within these intervals and running the model using the Euler scheme; (3) Identifying the optimal parameter set (reduced intervals) by iterating the process multiple times, evaluating the Nash-Sutcliffe Efficiency (NSE) score at each step, and comparing it with previous values. This iterative refinement gradually narrows the initial intervals, ensuring more reliable and well-optimized parameters for the model. Following these steps, we retained the optimal parameter sets μ∈[0.65,0.85] and λ∈[7,10] in the case of Bonou and μ∈[0.7,1] and λ∈[3,5] for Savè. To determine the best parameter values, we generated 10,000 random samples using different combinations of the parameters in the above ranges, ensuring optimal performance in this study.

The LSTM model was trained using the previous day’s discharge series as input. The same architecture and optimizer are used for both sub-catchments, with 80 hidden units and 300 epochs in Bonou, while only the number of hidden units (105) and epochs (250) are adjusted for Savè.

We implemented the hybridization of HyMoLAP with a Bayesian LSTM network, structured as an encoder-decoder architecture. The encoder processes the input sequence through a first LSTM layer, while the decoder refines the representation using a second LSTM layer before a fully connected layer maps the final output to the predicted streamflow. Monte Carlo dropout was applied to approximate the posterior distribution of the network’s weights, introducing stochasticity into the LSTM structure. The training process employed the Mean Squared Error (MSE) loss function, optimized with Adam at a learning rate of 0.01. In the Bonou sub-catchment, the first LSTM layer consistently contained 64 hidden units across all lead times, while the second layer varied depending on the forecast horizon: 64 hidden units for 1-day and 3-day lead times and 128 hidden units for 7-day and 10-day lead times. A dropout probability of 0.5 was applied in both LSTM layers to introduce stochasticity, and a batch size of 128 was used. The number of training epochs was adjusted based on the lead time, with 80 epochs for 1-day and 3-day lead times, 120 epochs for the 7-day lead time, and 250 epochs for the 10-day lead time. For the Savè sub-catchment, accurate forecasting was achieved by varying the number of hidden units and dropout probability depending on the forecast horizon. For the 1-day lead time, both the encoder and decoder layers contained 64 hidden units, trained over 120 epochs with a batch size of 256. For the 3-day lead time, we explored a configuration with 128 encoder units and 64 decoder units, using a dropout probability of 0.65, trained for 120 epochs with a batch size of 256. For the 7-day lead time, both the encoder and decoder contained 64 hidden units with a dropout probability of 0.7, trained over 120 epochs with the same batch size. For the 10-day lead time, the encoder had 128 hidden units, while the decoder contained 16 hidden units with a dropout probability of 0.5, trained over 120 epochs with a batch size of 256.

## 4 Results and discussions

This section presents and discusses the results in two parts. First, we compare the performance of the HyMoLAP-based approach with a LSTM model, followed by the results obtained through hybridization with Bayesian LSTM.

### 4.1 Prediction with HyMoLAP

**Fig 5:**
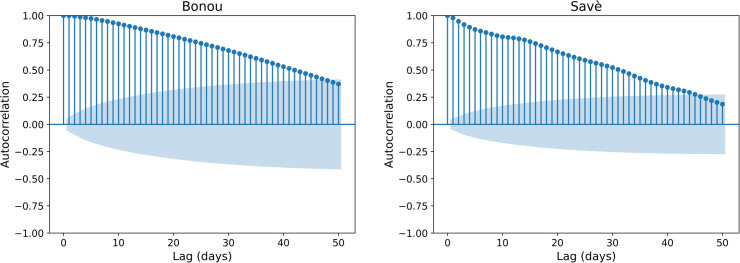
Autocorrelation function (ACF) of streamflow in Bonou and Savè sub-catchments.

The HyMoLAP model was first successfully calibrated to determine its optimal parameters. For the Bonou sub-catchment, the estimated parameters are μ=0.8001 and λ=8.2427, while for the Savè sub-catchment, they are μ=0.8888 and λ=3.9954. By applying the model given in Eq (15), simulations were first carried out for 1-day ahead prediction. To extend forecasts to multiple days, each new prediction was iteratively based on the previous ones. This is particularly relevant since streamflow data exhibit autocorrelation, as illustrated in Fig 5. The autocorrelation analysis indicates that, at the 95% confidence level, significant correlations persist even at long lags, which reflects both hydrological persistence and the large sample size where small correlations become statistically detectable. In our formulation for extended lead times, the Euler discretization was designed to capture local dependence between successive timesteps (${\hat{Q}}_{t-1},{\hat{Q}}_{t},{\hat{Q}}_{t+1}$). By iterating this relation, one can recover the extended correlations observed at higher lags, thus mitigating, though not necessarily eliminating, the necessity for additional explicit lag terms. This study presents the results for 1-, 3-, 7-, and 10-day-ahead streamflow predictions for the two sub-catchments.

**Fig 6:**
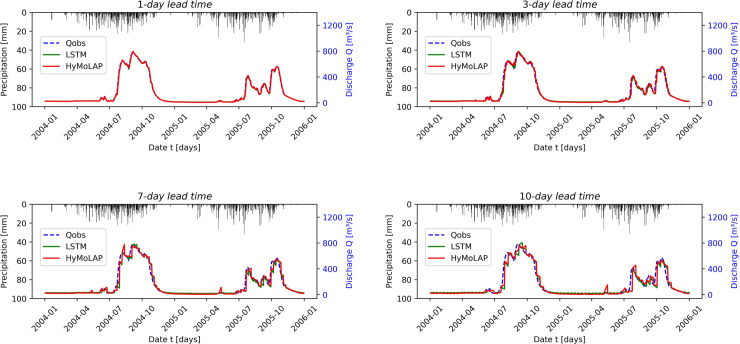
Comparison between observed and forecasted streamflow during the testing period using the HyMoLAP and LSTM models with different lead times in the Bonou sub-catchment.

Fig 6 presents the comparison between observed and forecasted streamflow using HyMoLAP and LSTM models during the testing period for different lead times in the Bonou sub-catchment. This graphical result is further explained by Tables 1 and 2, which provide the performance evaluation using the NSE, RMSE, and KGE metrics. We first observe a very strong performance of the HyMoLAP model in predicting one-day-ahead streamflow, as indicated by NSE and KGE values very close to 1. Furthermore, the performance decreases as the lead time increases, which is an expected outcome since no observed information is used during these periods. This trend is particularly reflected in the RMSE values, which increase from 12.567 for a 1-day lead time to 60.728 for a 10-day lead time. Despite this, the NSE value remains above 0.92 for the 10-day-ahead forecast. According to [113], this result indicates that the formulated approach demonstrates excellent performance in simulating multi-day-ahead streamflow in the Bonou sub-catchment. A similar observation can be made considering the KGE metric, which remains above 0.95 for all lead times considered, further confirming the model’s robustness. Moreover, compared to LSTM, the HyMoLAP-based approach demonstrates similar performance across all metrics and lead times, although the LSTM model exhibits a slight decline in accuracy, as shown in Tables 1 and 2. This consistency across different performance metrics suggests that the HyMoLAP model effectively captures streamflow dynamics, even for extended forecasting horizons in the Bonou sub-catchment.

**Table 1:** Performance of HyMoLAP in the Bonou and Savè sub-catchments for calibration and validation.

Phase	Lead time	Performance metrics					
		Bonou			Savè		
		NSE	RMSE	KGE	NSE	RMSE	KGE
**Calibration**							
HyMoLAP	1-day	0.997	13.486	0.998	0.964	44.561	0.980
	3-day	0.991	26.047	0.995	0.916	68.309	0.951
	7-day	0.967	48.787	0.981	0.824	98.826	0.861
	10-day	0.957	56.093	0.977	0.743	119.547	0.799
**Validation**							
HyMoLAP	1-day	0.997	12.567	0.998	0.970	37.140	0.981
	3-day	0.986	25.702	0.991	0.917	61.308	0.953
	7-day	0.955	45.895	0.977	0.799	95.609	0.900
	10-day	0.921	60.728	0.959	0.811	90.437	0.834

**Table 2:** Performance of LSTM in the Bonou and Savè sub-catchments for training and validation.

Phase	Lead time	Performance metrics					
		Bonou			Savè		
		NSE	RMSE	KGE	NSE	RMSE	KGE
**Training**							
LSTM	1-day	0.997	14.615	0.998	0.959	48.017	0.970
	3-day	0.988	29.199	0.994	0.892	77.543	0.934
	7-day	0.958	55.392	0.978	0.820	100.031	0.848
	10-day	0.936	68.089	0.968	0.749	118.182	0.816
**Validation**							
LSTM	1-day	0.996	13.800	0.992	0.966	39.437	0.967
	3-day	0.983	28.106	0.983	0.929	56.429	0.933
	7-day	0.946	50.069	0.961	0.899	67.856	0.903
	10-day	0.915	62.968	0.946	0.820	90.446	0.859

**Fig 7:**
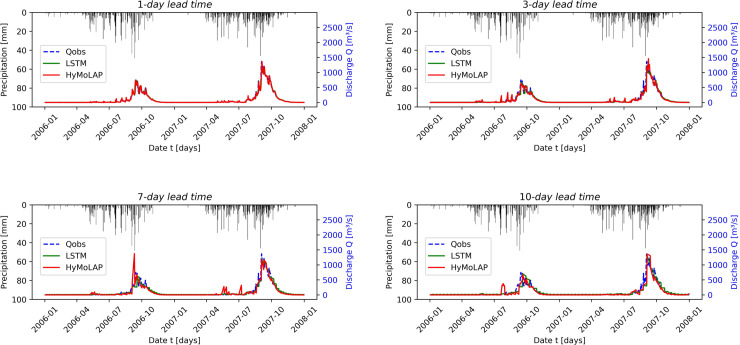
Comparison between observed and forecasted streamflow during the testing period using the HyMoLAP and LSTM models with different lead times in the Savè sub-catchment.

Fig 7 compares observed and simulated streamflow for different lead times during the testing phase in the Savè sub-catchment. This simulation exhibits greater variability than in the Bonou case, which can be attributed to the higher precipitation volume. Based on the evaluation metrics presented in Table 1, the HyMoLAP model again demonstrates excellent performance in simulating one-day-ahead streamflow, as indicated by an NSE of 0.970, further confirmed by the KGE. We also observe a performance decline, which occurs more rapidly than in the Bonou case, as the lead time increases. This is likely related to the smaller basin size of Savè (≈ 23,600 km^2^) compared to Bonou (≈ 48,900 km^2^), which increases the sensitivity of streamflow to local precipitation variability and makes multi-day predictions more challenging. However, the simulation quality remains very good across all lead times, as indicated by the NSE values. In this sub-catchment, HyMoLAP provides better predictions only for 1-day-ahead forecasts compared to the LSTM model (Table 2), although the performance difference between the two models remains slight across all lead times, except for the 7-day lead time, where the LSTM performs noticeably better than HyMoLAP, as indicated by the metrics. These results highlight that the HyMoLAP model is capable of simulating multi-day-ahead streamflow even in catchments with higher variability.

It is evident from the two figures (Figs 6 and 7) that some estimated peak values in the predicted time series fail to match the observed values, particularly for long lead times. This issue arises from the accumulation of prediction errors in our iterative forecasting approach, which amplifies fluctuations and generates artificial peaks, particularly in basins with strong variability such as Savè. This indicates that, compared to LSTM, the proposed HyMoLAP-based approach is more sensitive to error accumulation for large lead times. Nevertheless, the overall predictive performance of HyMoLAP remains comparable to that of LSTM according to the various statistical metrics across the two sub-catchments. This suggests that, despite its higher sensitivity to peak errors, HyMoLAP is as reliable as LSTM for capturing the overall streamflow dynamics across the two sub-catchments.

**Fig 8:**
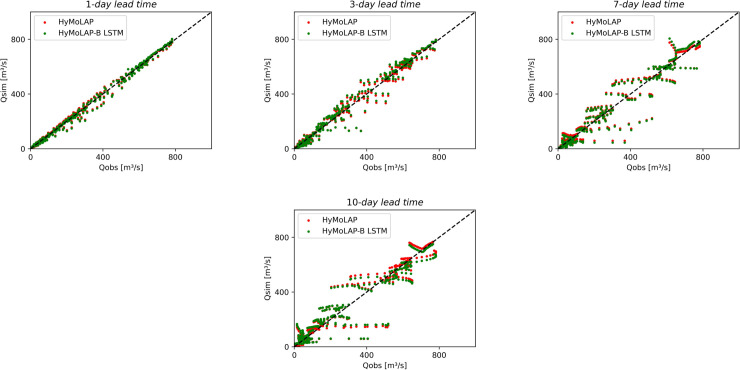
Scatter plots of observed vs. multi-step forecasted streamflow using HyMoLAP and the hybrid HyMoLAP–Bayesian LSTM in the Bonou sub-catchment.

**Fig 9:**
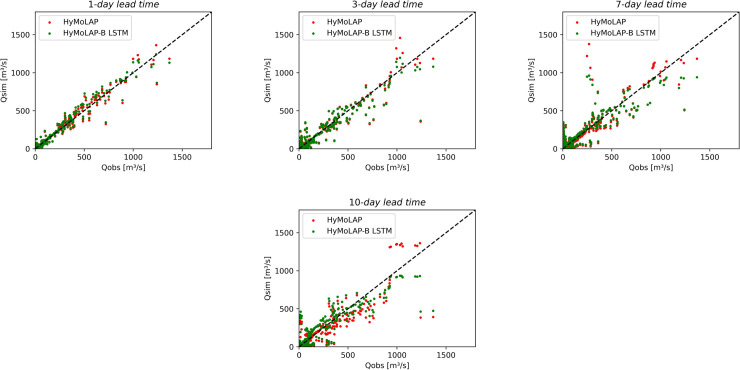
Scatter plots of observed vs multi-step forecasted streamflow using HyMoLAP and the hybrid HyMoLAP–Bayesian LSTM in the Savè sub-catchment.

From the comparison with the LSTM model and based on the performance metrics, the proposed HyMoLAP-based framework exhibits strong performance, particularly for short-term predictions, as observed in the 1-day and 3-day lead time simulations in both sub-catchments. Additionally, for short lead times, rapid fluctuations in streamflow data, especially those induced by climatic conditions, are less pronounced and do not significantly affect the modeling approach. As a result, the approach, like the LSTM network, yields better results for catchments with low noise in streamflow data, as evidenced in Bonou compared to the Savè sub-catchment. The next subsection builds upon the HyMoLAP-related results presented above by introducing and discussing interval predictions, as quantifying uncertainties is essential for probabilistic forecasting and risk assessment in hydrological modeling. The scatter plots of the HyMoLAP model can be observed in Figs 8 and 9, for both sub-catchments, illustrating how closely the predicted streamflow values align with observations across different lead times, thereby confirming the analysis conducted above.

### 4.2 Prediction with the hybrid HyMoLAP–Bayesian LSTM

The hyperparameters of the hybrid HyMoLAP–Bayesian LSTM model, including the number of hidden units, dropout probability, batch size, and number of training epochs, were selected through a combination of grid search and manual tuning based on model performance. We tested multiple configurations for each lead time and sub-catchment, and the settings reported in Experiment design Sect 3.7 correspond to those yielding the best prediction results. The primary objective of this study in developing this hybrid framework is to quantify uncertainty in the proposed multi-step forecasting approach. However, simulations have shown that this method can also improve the point predictions obtained with the HyMoLAP model.

To assess the performance of the hybrid HyMoLAP–Bayesian LSTM model in daily streamflow forecasting, we conducted a comparative analysis with the HyMoLAP model. As shown in Table 3, the proposed HyMoLAP–Bayesian LSTM exhibits similar performance to the HyMoLAP-based approach in terms of NSE and RMSE for all lead times in the Bonou sub-catchment (Table 1). However, the KGE for this hybrid model shows lower accuracy for the 7- and 10-day lead times, which can be attributed to differences in how correlation, bias, and variability are weighted in this metric. This observation is further confirmed by analyzing the scatter plots in Fig 8. It can be seen that both approaches yield a similar distribution of streamflow across all lead times, with the 1- and 3-day lead time results showing particularly close agreement between the two models. Nevertheless, the hybrid model remains competitive in the Bonou sub-catchment, demonstrating its ability to capture streamflow dynamics even for extended lead times.

**Table 3:** Performance of hybrid HyMoLAP–Bayesian LSTM (HyMoLAP-BLSTM) in the Bonou and Savè sub-catchments for training and validation.

Phase	Lead time	Performance metrics					
		Bonou			Savè		
		NSE	RMSE	KGE	NSE	RMSE	KGE
**Training**							
HyMoLAP-BLSTM	1-day	0.996	13.89	0.993	0.961	46.515	0.975
	3-day	0.990	27.283	0.9930	0.918	67.430	0.923
	7-day	0.963	51.543	0.908	0.838	95.006	0.870
	10-day	0.957	56.020	0.961	0.796	106.440	0.793
**Validation**							
HyMoLAP-BLSTM	1-day	0.996	13.221	0.996	0.968	38.146	0.980
	3-day	0.984	27.196	0.978	0.923	58.894	0.938
	7-day	0.953	47.040	0.945	0.833	86.952	0.819
	10-day	0.920	60.979	0.931	0.839	85.386	0.826

The evaluation metric results of the HyMoLAP–Bayesian LSTM hybrid model in the Savè sub-catchment, displayed in Table 3, indicate that the hybrid model performs similarly to the HyMoLAP model in simulating streamflow at 1- and 3-day lead times based on NSE and RMSE values, despite a slight difference in RMSE for the 3-day lead time. However, the most notable improvement is observed for longer lead times, with NSE increasing from 0.799 to 0.833 for the 7-day lead time and from 0.811 to 0.839 for the 10-day lead time, while RMSE decreases from 95.609 to 86.952 for the 7-day lead time and from 112.039 to 102.717 for the 10-day lead time. On the other hand, the KGE metric shows lower accuracy in this sub-catchment. These findings align with the scatter plots displayed in Fig 9, where increasing lead times result in more dispersed distributions. Moreover, for the 7- and 10-day forecasts, the predicted versus observed points tend to be closer to the line *y* = *x*, indicating a reduction in bias and reflecting an overall improvement in long-term streamflow predictions.

Many studies have explored this type of hybridization, integrating simulated processes or outputs from hydrological models into machine learning frameworks [44,114], demonstrating improved performance. The hybridization here results in a novel hybrid model with two advantages: (1) improving multi-step point predictions, as observed in the Savè sub-catchment, and (2) primarily enabling uncertainty quantification through Bayesian inference. In terms of deterministic prediction performance, both HyMoLAP and the hybrid HyMoLAP–Bayesian LSTM models provide strong predictions in both sub-catchments. Up to 10-day lead times, the performance remains very good (NSE equal to or greater than 0.8). The ability of the hybrid approach to quantify multi-step uncertainty in both sub-catchments, along with its capacity to improve accuracy in the Savè sub-catchment, suggests that further enhancements could be achieved by incorporating more advanced machine learning models into this framework. This ability of the HyMoLAP–Bayesian LSTM hybrid model to generate accurate results enhances confidence in the uncertainty quantification of predicted streamflow.

**Fig 10:**
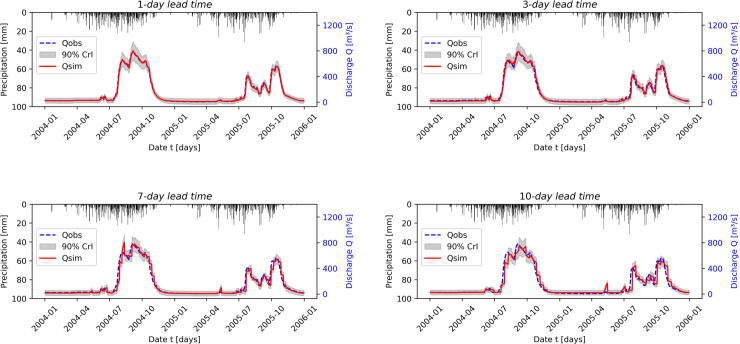
90% prediction intervals of the HyMoLAP-Bayesian LSTM hybrid model for uncertainty quantification during the testing period at different lead times in the Bonou sub-catchment.

**Fig 11:**
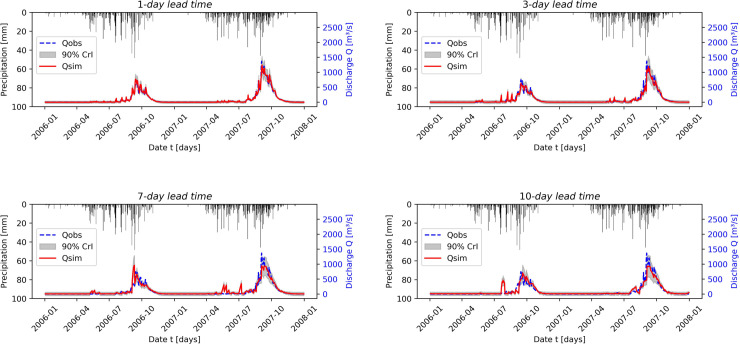
90% prediction intervals of the HyMoLAP-Bayesian LSTM hybrid model for uncertainty quantification during the testing period at different lead times in the Savè sub-catchment.

In addition to evaluating forecasting accuracy, we primarily focus on assessing forecast uncertainty. The uncertainty quantification by the HyMoLAP–Bayesian LSTM hybrid model is illustrated in Figs 10 and 11. The results are presented for various lead times in the Bonou and Savè sub-catchments. The red line represents the predicted streamflow by the hybrid model, whose performance evaluation is summarized in Table 3. The uncertainty is represented by prediction intervals computed at a 90% credible level. The 90% prediction interval is one of the most commonly used for streamflow uncertainty quantification, as demonstrated in [63,64,115,116]. We can observe that most observed streamflow points fall within the predicted intervals across all lead times in both sub-catchments. To quantitatively assess this observation, we used the Prediction Interval Coverage Probability (PICP) and the Mean Prediction Interval Width (MPIW).

**Fig 12:**
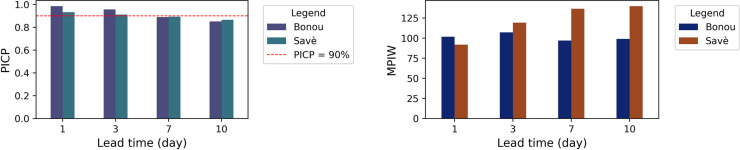
PICP and MPIW values for the 90% prediction intervals of HyMoLAP-Bayesian LSTM in uncertainty quantification (UQ) for the Bonou and Savè sub-catchments at different lead times.

The results of PICP and MPIW for both sub-catchments at different lead times are shown in Fig 12. As indicated in [63], for a 90% credible level, an ideal uncertainty quantification result is achieved when at least 90% of the observations fall within the prediction interval while maintaining a small MPIW value. It is evident from Fig 12 that the HyMoLAP–Bayesian LSTM hybrid model provides PICP values above 90% at 1- and 3-day lead times in the Bonou sub-catchment. However, at the 7- and 10-day lead time, the PICP does not reach 90% but remains close to this threshold. In this sub-catchment, the MPIW values range between 92 and 108 *m*^3^/*s* for all lead times. These results indicate strong confidence in the prediction intervals, particularly for 1-, 3-, and 7-day lead times. In the Savè sub-catchment, the PICP values also exceed 90% at 1- and 3-day lead times and remain very close to this level at 7- and 10-day lead times. A notable observation in this sub-catchment is the increasing MPIW as lead time increases, indicating higher uncertainty in predictions over longer lead times. This trend aligns with the results observed in point simulation, as reflected by the evaluation metrics for point prediction summarized in Table 3. At long lead times in both sub-catchments, although adjusting the dropout probability might further improve coverage, we tested various values of this parameter along with other hyperparameters, and the reported configuration consistently yielded the best trade-off between PICP and MPIW.

Based on the performance for uncertainty quantification in both sub-catchments, it is clear that the HyMoLAP–Bayesian LSTM hybrid model generates consistent prediction intervals in the Bonou and Savè sub-catchments. The calculated uncertainty can serve as a quantifier for forecast errors, which commonly signify the deviation between the forecasts and the observations [63,64]. A well-calibrated uncertainty quantification ensures that these deviations are properly accounted for, offering a probabilistic interpretation of forecast reliability. In both sub-catchments, the high PICP values indicate that the model effectively captures the variability of streamflow, providing confidence in predictions. However, the increase in MPIW, particularly in the Savè sub-catchment, highlights the growing uncertainty associated with longer lead times, which is expected due to the accumulation of errors in multi-step forecasting. These results emphasize the importance of integrating uncertainty quantification into hydrological forecasting to enhance decision-making. The ability of the HyMoLAP–Bayesian LSTM hybrid model to provide reliable prediction intervals suggests its potential for operational applications, particularly in water resource management and flood risk assessment, where understanding forecast confidence is as crucial as the point predictions themselves. The model also remains computationally efficient, requiring only moderate resources for real-time deployment, though adequate resources may be needed for large-scale or high-frequency forecasting. Further improvements, such as integrating physical or hydrological constraints, could refine uncertainty quantification and strengthen the reliability of the proposed multi-step streamflow forecasting framework across various catchment conditions.

## 5 Conclusion

In this study, we presented a novel framework for multi-step ahead streamflow forecasting by building upon the Hydrological Model based on the Least Action Principle (HyMoLAP). Compared to data-driven methods, hydrological models offer a structured foundation that can drive improvements by integrating physical constraints, enhancing interpretability, and enabling hybrid approaches that leverage both physics-based and data-driven strengths. Since HyMoLAP is formulated as an ordinary differential equation that models changes between consecutive streamflows, we used it for 1-day ahead forecasting and then derived a recurrent relation to entend predictions to multi-day ahead streamflow forecasting. To enable uncertainty quantification, we improved the framework by hybridizing it with a Bayesian LSTM model, employing Monte Carlo dropout as a variational approximation of the posterior distribution. The proposed framework was applied to the Bonou and Savè sub-catchments within the Ouémé River Basin, Benin. Regarding the performance of the LSTM model at different lead times, the results demonstrated that both the HyMoLAP-based approach and its hybrid variant integrating a Bayesian LSTM are capable of accurately simulating streamflow in the Bonou and Savè sub-catchments. They particularly demonstrate strong performance for short-term predictions, as observed for 1- and 3-day lead times in both sub-catchments. The hybridization with Bayesian LSTM provided a slight improvement in predictive accuracy, particularly in Savè at longer lead times, and delivered reliable uncertainty quantification across lead times in both sub-catchments. Nevertheless, performances decreased more sharply at extended horizons, especially in Savè where streamflow variability and noise are more pronounced. In particular in this sub-catchment, the framework tended to generate some artificial peaks, reflecting the difficulty of the recurrent formulation to capture long-term variations under noisy conditions. This sensitivity to hydrological variability contrasts with Bonou, where lower streamflow fluctuations allowed for more stable forecasts. Furthermore, the current version of HyMoLAP, like the GR4J model, does not include a snow component, which limits its direct applicability in snow-dominated basins.

The proposed methodology serves as an additional tool for multi-step streamflow forecasting in hydrology. Looking ahead, several extensions could further improve the framework. Incorporating physical or hydrological constraints into the HyMoLAP-based or hybrid framework may enhance accuracy and yield more precise uncertainty estimates, while also strengthening robustness across diverse catchment conditions. Refining the recurrent formulation derived from HyMoLAP could help mitigate artificial peaks and enhance the model’s long-term predictive stability. In addition, exploring alternative hybridization strategies, such as those employing Transformers or Kolmogorov–Arnold Networks (KAN), also represents a promising avenue. These potential avenues for improvement, combined with the demonstrated computational efficiency of the current approach, support its potential for operational hydrological forecasting under a wider range of conditions.

## Supporting information

S1 Data Data. (PDF)

S2 Document Bayesian neural networks and MC dropout. (PDF)
