# Supplementary material for: Multi-step ahead streamflow and uncertainty forecasting using a HyMoLAP rainfall-runoff model-based framework integrated with Bayesian neural networks in the Ouémé river basin, Benin
Source: PLoS One. 2025 Oct 7;20(10):e0333590. doi: 10.1371/journal.pone.0333590 (PMC12503268; doi:10.1371/journal.pone.0333590)
Supplement: S1 Data — (PDF) [file pone.0333590.s001.pdf]

S1 Data. Data

The time series data were obtained from Météo-Benin and the National Directorate of Water (DG-Eau), Benin. Due to institutional data-sharing policies, these datasets cannot be made freely accessible. The open-source code developed is available in the corresponding GitHub repository: `https://github.com/Ezesia-lab/HyMoLAP_Bayesian_LSTM`.
