# Supplementary material for: Multi-step ahead streamflow and uncertainty forecasting using a HyMoLAP rainfall-runoff model-based framework integrated with Bayesian neural networks in the Ouémé river basin, Benin
Source: PLoS One. 2025 Oct 7;20(10):e0333590. doi: 10.1371/journal.pone.0333590 (PMC12503268; doi:10.1371/journal.pone.0333590)
Supplement: S2 Document — (PDF) [file pone.0333590.s002.pdf]

S2 File. Bayesian Neural Networks and MC Dropout

Let θ represent the set of neural network parameters, which typically includes the weights and biases. Bayesian inference relies on Bayes' theorem to update a prior probability distribution based on new observations. The general formula is given by:

$$p(\theta \mid D) = \frac{p(D \mid \theta)p(\theta)}{p(D)}, \tag{1}$$

where $p(\theta \mid D)$ is the posterior distribution of the parameters θ after observing the data D, $p(D \mid \theta)$ is the likelihood of the data given θ, $p(\theta)$ is the prior distribution of the parameters, and $p(D)$ is the evidence, defined as:

$$p(D) = \int p(D \mid \theta)p(\theta)d\theta. \tag{2}$$

Equation (1) highlights that the parameters θ, particularly the weights W, are not fixed values but rather treated as random variables. Their prior distribution is updated as new data becomes available, resulting in a posterior distribution $p(\theta \mid D)$, which captures the uncertainty in the model's parameters.

MC Dropout constitutes a variational approximation of Bayesian inference. It is based on the assumption that each weight matrix W_i (now random), of dimension $K_i \times K_{i-1}$ for each layer i in the network, follows a variational distribution, defined as follows:

$$q(W_i) = M_i \cdot \text{diag}([z_{i,j}]_{j=1}^{K_i}), \tag{3}$$

where

$$z_{i,j} \sim \text{Bernoulli}(p_i), \quad \text{for } i = 1, 2, \ldots, L \text{ and } j = 1, 2, \ldots, K_{i-1}, \tag{4}$$

with M_i (deterministic matrix) representing the matrix of weights before dropout is applied, while $\text{diag}(z_{i,j})$ is a diagonal matrix whose elements $z_{i,j}$ are independent Bernoulli random variables with probability p_i. L is the total number of layer and the parameter p_i represents the probability of retaining a unit in layer i during dropout, referred to as the activation probability. It is related to the dropout probability d_i by $p_i = 1 - d_i$. This parameter can either be manually specified or learned during training.

The objective of training under this framework is to find a variational distribution $q(W)$ that minimizes the Kullback-Leibler (KL) divergence with the true posterior $p(W \mid D)$:

$$q^*(W) = \arg\min_{q(W)} \text{KL}(q(W) \parallel p(W \mid D)), \tag{5}$$

where the KL is defined as:

$$\text{KL}(q(W) \parallel p(W \mid D)) = \int q(W) \log \frac{q(W)}{p(W \mid D)} dW. \tag{6}$$

In other words, the goal is to optimize $q(W)$ so that it closely approximates the true posterior $p(W \mid D)$, which is the central principle of variational inference. The predictions of the model on a new test example x are then given by the Bayesian model average (BMA) as follows:

$$p(y \mid x, D) = \int p(y \mid x, W)p(W \mid D)dW, \tag{7}$$

where $p(y \mid x, W)$ is the predictive distribution for a given value of the parameters W. The integral (7) represents the expected predictive distribution by averaging over all possible weight configurations, weighted by their posterior probability.
